# Parisian ruin for the dual risk process in discrete-time

**DOI:** 10.1007/s13385-018-0172-8

**Published:** 2018-04-25

**Authors:** Zbigniew Palmowski, Lewis Ramsden, Apostolos D. Papaioannou

**Affiliations:** 1 Department of Applied Mathematics, Wrocław University of Science and Technology, Wrocław, Poland; 2 Institute for Financial and Actuarial Mathematics, Department of Mathematical Sciences, University of Liverpool, Liverpool, L69 7ZL United Kingdom

**Keywords:** Dual risk model, Discrete-time, Ruin probabilities, Parisian ruin, Binomial/geometric model, Parisian gambler’s ruin

## Abstract

In this paper we consider the Parisian ruin probabilities for the dual risk model in a discrete-time setting. By exploiting the strong Markov property of the risk process we derive a recursive expression for the finite-time Parisian ruin probability, in terms of classic discrete-time dual ruin probabilities. Moreover, we obtain an explicit expression for the corresponding infinite-time Parisian ruin probability as a limiting case. In order to obtain more analytic results, we employ a conditioning argument and derive a new expression for the classic infinite-time ruin probability in the dual risk model and hence, an alternative form of the infinite-time Parisian ruin probability. Finally, we explore some interesting special cases, including the binomial/geometric model, and obtain a simple expression for the Parisian ruin probability of the gambler’s ruin problem.

## Introduction

The compound binomial model, first proposed by Gerber [17], is a discrete-time analogue of the classic Cramér–Lundberg risk model which provides a more realistic analysis to the cash flows of an insurance firm. The model has attracted attention since its introduction due to the recursive nature of the results, which are readily programmable in practise, and as a tool to approximate the continuous-time risk model as a limiting case (for details see [15]). In the compound binomial risk model, it is assumed that income is received via a periodic premium of size one, whilst the initial reserve and the claim amounts are assumed to be integer valued. That is, the reserve process of an insurer, denoted $\{R_n\}_{n \in \mathbb {N}}$, is given by

1.1 $$\begin{aligned} R_n=u+n-X_n, \end{aligned}$$

where $u\in \mathbb {N}$ is the insurers initial reserve and

1.2 $$\begin{aligned} X_n=\sum _{i=1}^n Y_i, \quad n=1,2,3, \ldots , \end{aligned}$$

denotes the aggregate claim amount up to period $n\in \mathbb {N}$, with $X_0=0$. Further, it is assumed that the random non-negative claim amounts, namely $Y_i$, $i=1,2,\ldots$, are independent and identically distributed (i.i.d.) random variables with probability mass function (p.m.f.) $p_k=\mathbb {P}(Y=k)$, for $k=0,1,2, \ldots$, and finite mean $\mathbb {E}(Y_1)<\infty$. We point out, due to its importance in the following, that the claim amounts $Y_i$, $i=1,2,\ldots$ have a mass point at zero with probability $p_0>0$.

Let *T* denote the time to ruin for the discrete-time risk model given in Eq. (1.1), defined by

$$\begin{aligned} T=\inf \{n\in \mathbb {N}: R_n \leqslant 0\}, \end{aligned}$$

where $T=\infty$ if $R_n>0$ for all $n\in \mathbb {N}$. Note that this definition is consistent with Gerber [17], whilst other authors define the ruin time when the reserve takes strictly negative values (see e.g. [28]). Then, the finite-time ruin probability, from initial reserve $u \in \mathbb {N}$, is defined by

$$\begin{aligned} \psi (u,t)=\mathbb {P}(T< t \, \big | R_0=u), \qquad t\in \mathbb {N}, \end{aligned}$$

with corresponding finite-time survival probability $\phi (u,t)=1-\psi (u,t)$. The finite-time ruin probability of the discrete-time risk model was first studied in [28], where explicit formulas are derived using generating functions. Later, Lefèvre and Loisel [18] derive a Seal-type formula based on the ballot theorem (see [27]) and a Picard–Lefèvre-type formula for the corresponding finite-time survival probability, namely $\phi (u,t)$. For further results on finite-time probabilities, see [19] and references therein. The finite-time ruin probabilities, in general, prove difficult to tackle and the literature on the subject remains few.

On the other hand, the infinite-time ruin probability, defined as the limiting case, i.e. $\psi (u)=\lim _{t\rightarrow \infty } \psi (u,t)$, has been considered by several authors, e.g. Gerber [17], Michel [23], Shiu [26] and Dickson [15], among others, where numerous alternative methods have been employed to derive explicit expressions. Further references for related results such as; the discounted probability of ruin, the deficit and surplus prior to ruin and the well known Gerber–Shiu function, to name a few, can be found in Cheng et al. [7], Cossette et al. [10, 11], Boudreault et al. [6], Dickson [15], Li and Garrido [20], Pavlova and Willmot [25], Wu and Li [29] and Yuen and Guo [30]. For a full comprehensive review of the discrete-time literature refer to Li et al. [21], and references therein.

One limitation of the discrete-time risk model (1.1), as pointed out by Avanzi et al. [4], is that depending on the line of business there are companies which are subject to a constant flow of expenses and receive income/gains as random events. For instance, pharmaceutical or petroleum companies, where the random gains come from new invention or discoveries, require an alternative to the compound binomial risk model such that the reserve process, namely $\{R^*_n\}_{n\in \mathbb {N}}$, is defined by

1.3 $$\begin{aligned} R^*_n=u-n+X_n, \end{aligned}$$

where $\{X_k\}_{k\in \mathbb {N}^+}$ has the same form as Eq. (1.2). This model is known as the *discrete-time dual risk model*. The continuous analogue of the dual risk model has been considered by various authors, with the majority of focus in dividend problems (see [4, 5, 9, 24] and references therein). Additionally, Albrecher et al. [1] considered the continuous-time dual risk model under a loss-carry forward tax system, where, in the case of exponentially distributed jump sizes, the infinite-time ruin probability is derived in terms of the ruin probability without taxation. However, the dual risk problem in discrete-time remains to be studied.

For convenience, throughout the remainder of this paper, we use the notation $\mathbb {P}(\cdot \,| R^*_0=u) = \mathbb {P}_u(\cdot )$ and $\mathbb {P}_0(\cdot )=\mathbb {P}(\cdot )$.

The finite-time ruin probability, for the dual risk process given in Eq. (1.3), is defined in a similar way to the discrete-time risk model defined in Eq. (1.1). That is, the finite-time ruin probability is defined as the probability that the risk reserve process $\{R^*_n\}_{n \in \mathbb {N}}$ attains a non-positive level before some pre-specified time horizon $t\in \mathbb {N}$, from initial capital $u \in \mathbb {N}$. Since the reserve process for the dual risk model, defined in Eq. (1.3), experiences deterministic losses of one per period, it follows that the probability of experiencing a non-positive level is equivalent to the probability of hitting the zero level. Thus, let us denote the time to ruin for the dual risk model, given in Eq. (1.3), by $\tau ^*$, defined by

$$\begin{aligned} \tau ^*=\inf \{ n \in \mathbb {N} : R^*_n=0 \}. \end{aligned}$$

Then, the finite-time dual ruin probability is given by

1.4 $$\begin{aligned} \psi ^*(u,t)=\mathbb {P}(\tau ^* < t\, \big | R^*_0=u), \end{aligned}$$

with $\psi ^*(0,t)=1$, for all $t\in \mathbb {N}^+$.

The infinite-time dual ruin (survival) probability, as above, is defined as the limiting case, i.e. $\psi ^*(u)=\lim _{t\rightarrow \infty } \psi ^*(u,t)$. It is clear that $\tau ^* \geqslant u$ (due to the deterministic losses of one per period). Finally, it is assumed that the net profit condition holds, i.e. $\mu =\mathbb {E}(Y_1)>1$, such that $R^*_n \rightarrow +\infty$ as $n\rightarrow \infty$. This condition ensures that the dual ruin probability is not certain.

The aim of this paper is to extend the notion of ruin to the so-called Parisian ruin, which occurs if the process $\{R^*_n\}_{n\in \mathbb {N}}$ is strictly negative for a fixed number of periods $r \in \{1,2, \ldots \}$ and derive recursive and explicit expressions for the Parisian ruin probability in finite and infinite-time. The idea of Parisian ruin follows from Parisian stock options, where prices are activated or cancelled when underlying assets stay above or below a barrier long enough (see [8, 14]). The time of Parisian ruin, in the discrete-time dual risk model, is defined as

$$\begin{aligned} \tau ^r = \inf \{ n \in \mathbb {N} : n - \sup \{ s< n : R^*_s = -1, R^*_{s-1}=0 \}=r \in \mathbb {N}^+, \, R^*_n < 0 \}, \end{aligned}$$

with finite and infinite-time Parisian ruin probabilities defined by

$$\begin{aligned} \psi ^*_r(u,t)=\mathbb {P}_u(\tau ^r < t), \end{aligned}$$

and

$$\begin{aligned} \psi ^*_r(u)=\lim _{t\rightarrow \infty } \psi ^*_r(u,t), \end{aligned}$$

respectively. We further define the corresponding finite and infinite-time Parisian survival probabilities by $\phi ^*_r(u,t)= \mathbb {P}_u(\tau ^r \geqslant t)=1-\psi ^*_r(u,t)$ and $\phi ^*_r(u)= 1-\psi ^*_r(u)$.

The extension from classical ruin to Parisian ruin was first proposed, in a continuous time setting, by Dassios and Wu [14] for the compound Poisson risk process with exponential claim sizes. In this setting they derive expressions for the Laplace transform of the time and probability of Parisian ruin. Further, Czarna and Palmolski [12] and Loeffen et al. [22] have derived results for the Parisian ruin in the more general case of spectrally negative Lévy processes. More recently, Czarna et al. [13] adapted the Parisian ruin problem to a discrete-time risk model, as in Eq. (1.1), where finite and infinite-time expressions for the ruin probability are derived, along with the light and heavy-tailed asymptotic behaviour.

The paper is organised as follows: In Sect. 2, we exploit the strong Markov property of the risk process to derive a recursive formula for the finite-time Parisian ruin probability, with general initial reserve, in terms of the dual ruin probability defined in Eq. (1.4) and the Parisian ruin probability with zero initial reserve. For the latter risk quantity, we show this can be calculated recursively. In Sect. 3, we obtain a similar expression for the corresponding infinite-time Parisian ruin probability, where the Parisian ruin probability with zero initial reserve has an explicit form. In Sect. 4, we consider an alternative method for calculating the infinite-time dual ruin probability. In Sect. 5.1, in order to illustrate the applicability of our recursive type equation, we analyse the Binomial/Geometric model, as a special case. Finally, in Sect. 5.2, we derive an explicit expression for the Parisian ruin probability to the well known gambler’s ruin problem.

## Finite-time Parisian ruin probability

In this section, we derive an expression for the finite-time Parisian survival probability, $\phi ^*_r(u,t)$, for the dual risk model given in Eq. (1.3), for general initial reserve $u \in \mathbb {N}$.

First note that, since the dual risk process, $\{R^*_n\}_{n\in \mathbb {N}}$, experiences only positive random gains and losses occur at a rate of one per period, it follows that $\phi ^*_r(u,t)=1$, when $t \leqslant u + r +1$. Now, for $t > u + r +1$, by conditioning on the time to ruin, namely $\tau ^*$, using the strong Markov property and the fact that $\mathbb {P}_u(\tau ^* = k)=0$ for $k< u$, we have

2.1 $$\begin{aligned} \phi ^*_r(u,t)=\sum _{k=u}^{t-r-2} \mathbb {P}_u(\tau ^* =k)\phi ^*_r(0,t-k) + \phi ^*(u,t-r-1). \end{aligned}$$

Note that the finite-time dual survival probability is given by $\phi ^*(u,t)=1-\psi ^*(u,t)=1-\sum _{k=0}^{t-1} \mathbb {P}_u(\tau ^*=k)$. Thus, from the form of Eq. (2.1), in order to obtain an expression for the Parisian survival probability, $\phi ^*_r(u,t)$, we need only to derive expressions for $\mathbb {P}_u(\tau ^* =k)$ and the Parisian survival probability with zero initial reserve, namely $\phi ^*_r(0,t)$.

### Lemma 1

*In the discrete-time dual risk model, the probability of hitting the zero level from initial capital* $u\in \mathbb {N}$*, in* $n \in \mathbb {N}$ *periods, namely* $\mathbb {P}_u(\tau ^*=n)$*, is given by*

2.2 $$\begin{aligned} \mathbb {P}_u(\tau ^*=n) = \frac{u}{n}p_{n-u}^{*n}, \quad n \geqslant u, \end{aligned}$$

*where* $\{ p_k^{*n} \}_{n \in \mathbb {N}}$ *denotes the nth fold convolution of* $Y_1$.

### Proof

Consider the discrete-time dual risk process $\{R^*_n\}_{n\in \mathbb {N}}$, defined in Eq. (1.3), where

**Fig. 1:**
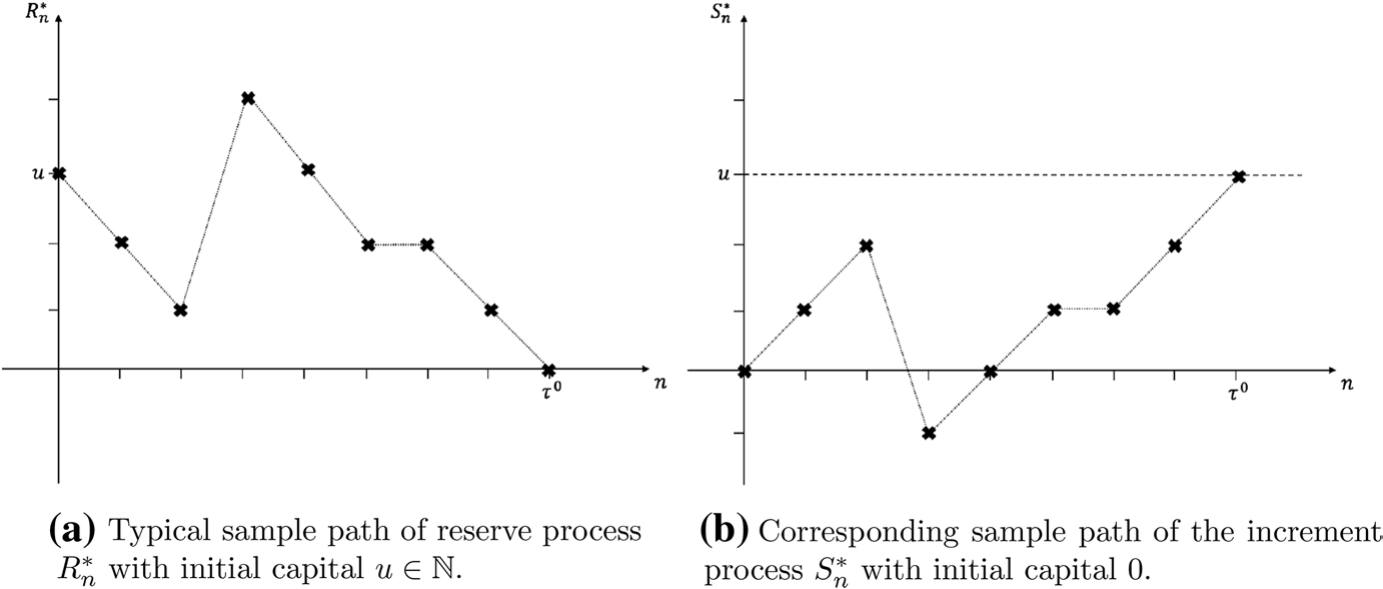
Equivalence between dual risk process and classic risk process

2.3 $$\begin{aligned} R^*_n=u-S^*_n, \end{aligned}$$

with $S^*_n=n-X_n$. The ‘increment’ process, $\{S^*_n\}_{n\in \mathbb {N}}$, is equivalent to a discrete-time risk process, given by Eq. (1.1), with initial capital $S^*_0=0$. Therefore, it follows that the dual ruin time, $\tau ^*$, is equivalent to the hitting time for the incremental process, $\{S^*_n\}_{n\in \mathbb {N}}$, of the level $u \in \mathbb {N}$ (see Fig. 1). Using Proposition 3.1 of Li and Sendova [19], the result follows. $\square$

Now that we have an expression for $\mathbb {P}_u(\tau ^*=k)$, $k\in \mathbb {N}$, and consequently for the finite-time dual survival probability, namely $\phi ^*(u,t)$, it remains to derive an expression for the finite-time Parisian survival probability for the case where the initial reserve is zero, i.e. $R^*_0=0$. Before we begin with deriving an expression for $\phi ^*_r(0,t)$, note that in order to avoid Parisian ruin, once the reserve process becomes negative, it will be necessary to return to the zero level (or above) in *r* time periods or less. Considering this observation, we will introduce another random stopping time, which we name ‘recovery’ time, that measures the number of periods it takes to recover from a deficit to a non-negative reserve. Let us denote the recovery time by $\tau ^-$, defined by

$$\begin{aligned} \tau ^-=\inf \{ n \in \mathbb {N} : R^*_n \geqslant 0, R^*_s< 0 , \;\;\forall s < n \}. \end{aligned}$$

Now, consider the dual risk reserve process defined in Eq. (1.3), with initial capital $u=0$. If no gain occurs in the first period of time, the risk reserve becomes $R^*_1=-1$ at the end of the period. On the other hand, if there is a random gain of amount $k\in \mathbb {N}^+$ in the first period, the risk reserve becomes $R^*_1=k-1$. Hence, by the law of total probability, we obtain a recursive equation for the finite-time Parisian survival probability, with initial capital zero, i.e. $\phi ^*_r(0,n)$ (where $n>r+1$), of the form

2.4 $$\begin{aligned} \phi ^*_r(0,n)&=p_0\, \phi ^*_r(-1, n-1)+ \sum _{k=1}^{\infty } p_k \phi ^*_r(k-1,n-1) \nonumber \\&=p_0 \sum _{s=1}^r \sum _{z=0}^{\infty } \mathbb {P}_{-1}(\tau ^- = s, R^*_{\tau ^-}=z)\phi ^*_r(z,n-s-1)\nonumber \\&\quad +\,\sum _{k=0}^{\infty } p_{k+1} \phi ^*_r(k,n-1), \end{aligned}$$

where $\mathbb {P}_{-1}(\tau ^- = \cdot , R^*_{\tau ^-}=\cdot )$ is the joint probability of the recovery time and the size of the overshoot at recovery, given initial capital $u=-1$.

In order to complete the above expression for $\phi ^*_r(0,n)$, we need first to derive an expression for $\mathbb {P}_{-1}(\tau ^- = \cdot , R^*_{\tau ^-}=\cdot )$, which is given in the following lemma.

### Lemma 2

*For,* $n\in \mathbb {N}^+$ *and* $k\in \mathbb {N}$ *, the joint distribution of the recovery time and the overshoot at recovery is given by*

2.5 $$\begin{aligned} \mathbb {P}_{-1}(\tau ^- = n, R^*_{\tau ^-}=k)&= \sum _{j=0}^{n-1}\, p_j^{*(n-1)}p_{1+n-j+k} -\sum _{j=2}^{n-1} \sum _{i=2}^j \frac{n-j}{n-i} p_{j-i}^{*(n-i)}\nonumber \\&\quad \times \,p_i^{*(i-1)} p_{1+n-j+k}, \end{aligned}$$

### Proof

**Fig. 2:**
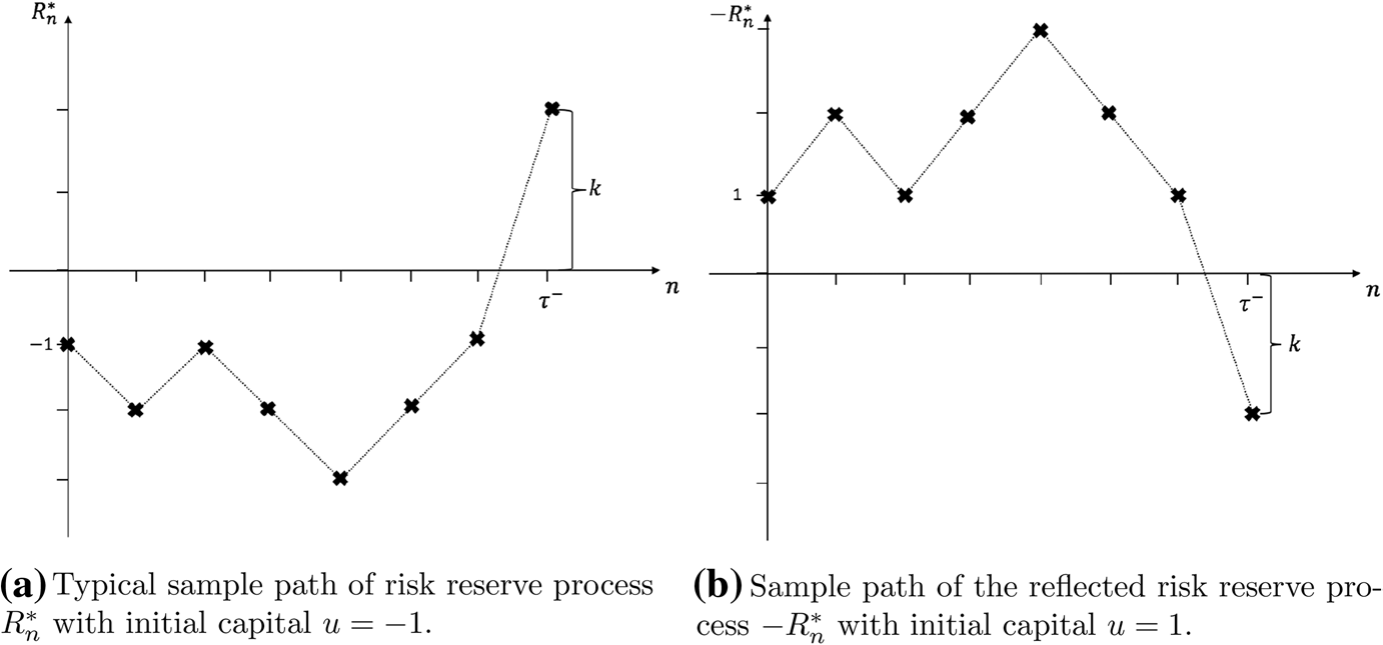
Equivalence between original and reflected risk processes

Consider the reflected discrete-time dual risk process, $\{-R^*_n\}_{n \in \mathbb {N}}$, where $\{R^*_n\}_{n \in \mathbb {N}}$ is given in Eq. (1.3), with initial capital $u=-1$. Then, it follows that the distribution of the time to cross the time axis and the overshoot of the process at this hitting time are equivalent for both $\{R^*_n\}_{n \in \mathbb {N}}$ and its reflected process $\{-R^*_n\}_{n \in \mathbb {N}}$, which can be described by a discrete-time risk process given in Eq. (1.1) (see Fig. 2). Thus, the joint distribution $\mathbb {P}_{-1}(\tau ^- = n, R^*_{\tau ^-}=k)$ can be found by employing the discrete ruin related quantity from Lemma 2 of [13]. That is, by setting $u=1$ in Eq. (4) of [13], the result follows. $\square$

Finally, substituting the form of $\phi ^*_r(u,t)$, given in Eq. (2.1), into Eq. (2.4), we obtain an expression for $\phi ^*_r(0,n)$, of the form

2.6 $$\begin{aligned} \phi ^*_r(0,n)&=p_0\sum _{s=1}^r \sum _{z=0}^\infty \mathbb {P}_{-1}(\tau ^-=s, R^*_{\tau ^-}=z) \phi ^*(z, n-s-r-2) \nonumber \\&\quad +\,p_0\sum _{s=1}^r \sum _{z=0}^\infty \sum _{i=z}^{n-s-r-3}\mathbb {P}_{-1}(\tau ^-=s, R^*_{\tau ^-}=z)\nonumber \\&\quad \times \,\mathbb {P}_z(\tau ^*=i) \phi ^*_r(0, n-s-i-1) \nonumber \\&\quad +\,\sum _{k=0}^\infty p_{k+1} \phi ^*(k, n-r-2) + \sum _{k=0}^\infty \sum _{i=k}^{n-r-3} p_{k+1} \mathbb {P}_k(\tau ^*=i)\nonumber \\&\quad \times \,\phi ^*_r(0, n-i-1). \end{aligned}$$

### Remark 1

An explicit expression for $\phi ^*_r(0,n)$, based on Eq. (2.6), proves difficult to obtain. However, due to the form of Eq. (2.6), a recursive calculation for $\phi ^*_r(0,n)$ is given by the following algorithm:

**Step 1** For $n=r+2$, in Eq. (2.6), and using the fact that $\phi ^*(u,t)=1$ for $t\leqslant u$, we have that

$$\begin{aligned} \phi ^*_r(0,r+2)&=p_0\sum _{s=1}^r \sum _{z=0}^\infty \mathbb {P}_{-1}(\tau ^-=s, R^*_{\tau ^-}=z)+1-p_0\\&=1-p_0\left( 1- \sum _{z=0}^\infty \mathbb {P}_{-1}(\tau ^- \leqslant r, R^*_{\tau ^-}=z)\right) \\&=1-p_0\phi (1,r+1), \end{aligned}$$

where $\phi (u,t)$ is the classic finite-time survival probability in the compound binomial risk model, which has been extensively studied in the literature, (see [19] and references therein) and alternatively can be evaluated using Lemma 2.

**Step 2** Based on the result of step 1, we can compute the following term, i.e. for $n=r+3$, we have

$$\begin{aligned} \phi ^*_r(0,r+3)&=p_0\sum _{s=1}^r \sum _{z=0}^\infty \mathbb {P}_{-1}(\tau ^-=s, R^*_{\tau ^-}=z)+\sum _{k=1}^\infty p_{k+1}+p_1\phi ^*_r(0,r+2)\\&=1-(1+p_1)p_0\phi (1,r+1). \end{aligned}$$

**Step 3** For $n=r+4$, we have

$$\begin{aligned} \phi ^*_r(0,r+4)&=p_0\left( \sum _{z=1}^\infty \mathbb {P}_{-1}(\tau ^-=1, R^*_{\tau ^-}=z)+\sum _{s=2}^r \sum _{z=0}^\infty \mathbb {P}_{-1}(\tau ^-=s, R^*_{\tau ^-}=z)\right) \\&\quad +\,p_0\mathbb {P}_{-1}(\tau ^-=1, R^*_{\tau ^-}=0)\phi ^*_r(0,r+2)+p_2\phi ^*(1,2)\\&\quad +\,\sum _{k=2}^\infty p_{k+1} +p_1\phi ^*_r(0,r+3)+p_2\mathbb {P}_1(\tau ^*=1)\phi ^*_r(0,r+2)\\&=p_0\left( \psi (1,r+1)-\mathbb {P}_{-1}(\tau ^-=1, R^*_{\tau ^-}=0)\right) \\&\quad +\,p_0\mathbb {P}_{-1}(\tau ^-=1, R^*_{\tau ^-}=0)\phi ^*_r(0,r+2)+p_2\left( 1-p_0\right) \\&\quad +\,1-(p_0+p_1+p_2)+p_1\phi ^*_r(0,r+3)+p_2p_0\phi ^*_r(0,r+2). \end{aligned}$$

Employing the results of steps 1 and 2 and using the fact that $\mathbb {P}_{-1}(\tau ^-=1, R^*_{\tau ^-}=0)=p_2$, by Lemma 2, after some algebraic manipulations we obtain

$$\begin{aligned} \phi ^*_r(0,r+4)&=1-\left[ 1+2p_0p_2+p_1(1+p_1)\right] p_0\phi (1,r+1). \end{aligned}$$

Thus, based on the above steps, it can be seen that $\phi _r^*(0,r+k)$, for $k=2,3,\ldots$, can be evaluated recursively for each value of *k* in terms of the mass functions, $p_k$, and the classic ruin quantity $\phi (1,r+1)$.

### Theorem 1

*For* $u\in \mathbb {N}$*, the finite-time Parisian ruin probability* $\psi ^*_r(u,t)=0$ *for* $t\leqslant u+r+1$ *and for* $t>u+r+1$*, is given by*

2.7 $$\begin{aligned} \psi ^*_r(u,t)=\sum _{k=u}^{t-r-2} \mathbb {P}_u(\tau ^* =k)\psi ^*_r(0,t-k), \end{aligned}$$

*where* $\mathbb {P}_u(\tau ^* =k)$ *is given in Lemma* 1 *and the initial value* $\psi ^*_r(0,n)$ *can be found recursively from Eq. *(2.6).

In the next subsection, we use the above expressions to derive results for the infinite-time Parisian ruin probabilities, for which, as will be seen, a more analytic expression can be found.

## Infinite-time Parisian ruin probability

In this section we derive an explicit expression for the infinite-time Parisian survival (ruin) probabilities using the arguments of the previous section. First, let us recall that the infinite-time Parisian survival probability is defined as $\phi _r^*(u)=\lim _{t\rightarrow \infty } \phi _r^*(u,t)$, with the infinite-time dual ruin quantities being defined in a similar way, i.e. $\phi ^*(u)=\lim _{t\rightarrow \infty } \phi ^*(u,t)$. Then, it follows by taking the limit $t\rightarrow \infty$, with $t \in \mathbb {N}$, Eq. (2.1) reduces to

3.1 $$\begin{aligned} \phi ^*_r(u) = \psi ^*(u)\phi ^*_r(0)+ \phi ^*(u), \end{aligned}$$

where $\phi ^*_r(0)$ is the infinite-time probability of Parisian survival with zero initial reserve and satisfies $\phi ^*_r(0)=\lim _{t \rightarrow \infty } \phi ^*_r(0,t)$, where $\phi ^*_r(0,t)$ is given by Eq. (2.4). Thus, $\phi ^*_r(0)$ is given by

$$\begin{aligned} \phi ^*_r(0) = p_0 \sum _{z=0}^\infty \mathbb {P}_{-1} (\tau ^- \leqslant r, R^*_{\tau ^-}=z) \phi ^*_r(z) + \sum _{j=0}^\infty p_{j+1} \phi ^*_r(j), \end{aligned}$$

or equivalently

3.2 $$\begin{aligned} \phi ^*_r(0) = \sum _{k=0}^\infty \left( p_0 \mathbb {P}_{-1} (\tau ^- \leqslant r, R^*_{\tau ^-}=k) + p_{k+1} \right) \phi ^*_r(k), \end{aligned}$$

where $\mathbb {P}_{-1} (\tau ^- \leqslant r, R^*_{\tau ^-}=k)$ can be obtained from the result of Lemma 2, i.e.

$$\begin{aligned} \mathbb {P}_{-1} (\tau ^- \leqslant r, R^*_{\tau ^-}=k)=\sum _{s=1}^r \mathbb {P}_{-1} (\tau ^- = s, R^*_{\tau ^-}=k). \end{aligned}$$

Considering the first term of the summation in the right hand side of Eq. (3.2) and solving with respect to $\phi ^*_r(0)$, we get an explicit representation for $\phi ^*_r(0)$, given by

3.3 $$\begin{aligned} \phi ^*_r(0) =C^{-1}\sum _{k=1}^\infty \left( p_0 \mathbb {P}_{-1} (\tau ^- \leqslant r, R^*_{\tau ^-}=k) + p_{k+1} \right) \phi ^*_r(k), \end{aligned}$$

where

$$\begin{aligned} C=1-p_0 \mathbb {P}_{-1}(\tau ^- \leqslant r, R^*_{\tau ^-}=0)-p_1. \end{aligned}$$

Now, since from Lemma 2 we can obtain an expression for the joint distribution of the time of recovery and the overshoot, namely $\mathbb {P}_{-1} (\tau ^- \leqslant r, R^*_{\tau ^-}=k)$, we can re-write Eq. (3.3) as

$$\begin{aligned} \phi ^*_r(0) =C^{-1}\sum _{k=1}^\infty a_k \phi ^*_r(k), \end{aligned}$$

where $a_k = \left( p_0 \mathbb {P}_{-1} (\tau ^- \leqslant r, R^*_{\tau ^-}=k) + p_{k+1} \right)$. Then, by substituting the general form of the infinite-time Parisian survival probability, given by (3.1), into the above equation, and solving the resulting equation with respect to $\phi ^*_r(0)$, we obtain

3.4 $$\begin{aligned} \phi ^*_r(0) =\frac{C^{-1}\sum _{k=1}^\infty a_k \phi ^*(k)}{1-C^{-1}\sum _{k=1}^\infty a_k \psi ^*(k)}. \end{aligned}$$

Note that, unlike for the finite-time case, in the infinite-time case we obtain an explicit expression for the Parisian survival probability, with zero initial reserve, which is given in terms of the infinite-time dual ruin probabilities. Thus, employing Eq. (3.1) and the result from Lemma 1 we obtain an explicit expression for the infinite-time Parisian survival probability, with general initial reserve $u\in \mathbb {N}$, given in the following theorem.

### Theorem 2

*For* $u\in \mathbb {N}$ *, the infinite-time Parisian ruin probability* $\psi ^*_r(u)$ *,is given by*

3.5 $$\begin{aligned} \psi ^*_r(u)=\psi ^*(u)\left( 1-\frac{C^{-1}\sum _{k=1}^\infty a_k \phi ^*(k)}{1-C^{-1}\sum _{k=1}^\infty a_k \psi ^*(k)}\right) , \end{aligned}$$

*where*

3.6 $$\begin{aligned} a_k= \left( p_0 \mathbb {P}_{-1} (\tau ^- \leqslant r, R^*_{\tau ^-}=k) + p_{k+1} \right) , \end{aligned}$$

*and*

$$\begin{aligned} C^{-1}=\left( 1-p_0 \mathbb {P}_{-1}(\tau ^- \leqslant r, R^*_{\tau ^-}=0)-p_1\right) ^{-1}. \end{aligned}$$

### Proof

The result follows by combining Eqs. (3.1) and (3.4), and recalling that $\phi ^*_r(u)=1-\psi ^*_r(u)$. $\square$

Note that in the above expression, for the infinite-time Parisian ruin probability $\psi ^*_r(u)$, all quantities are explicitly available by Lemma 1, Lemma 2 and the fact that $\psi ^*(u)=\sum _{k=0}^\infty \mathbb {P}_u(\tau ^*=k)$.

## An alternative approach to the infinite-time dual ruin probability

In Lemma 1, we obtained an expression for the probability function of the dual ruin time, namely $\mathbb {P}_u(\tau ^*=n)$, in terms of convolutions of the claim size distribution. This result, as discussed previously, can be used to obtain an expression for both the finite-time dual ruin probability and consequently, the infinite-time ruin probability, i.e.

$$\begin{aligned} \psi ^*(u)&=\sum _{k=0}^\infty \mathbb {P}_u(\tau ^*=k)= \sum _{k=u}^\infty \frac{u}{k}p_{k-u}^{*k}. \end{aligned}$$

Although the above expression is explicit, this representation does not provide much insight into the behaviour of the dual ruin probability, for which a closed form expression would be more favourable.

In this section, we consider an alternative derivation based on the fact that the ruin probability, $\psi ^*(u)$, satisfies a difference equation, for which a particular form of the solution is adopted. In the following, we show that this solution is indeed an analytical solution for $\psi ^*(u)$ and is unique. We point out that the following result can also be obtained using classical approaches, such as exponential martingales for random walks or the exponential change of measure (see [3]). However, these methods are more complex and require a deeper analysis than the proposed formulation.

Consider the dual risk reserve process given in Eq. (1.3) with initial reserve $u+1$, $u\in \mathbb {N}$ and condition on the possible events in the first time period. Then, by law of total probability, we obtain a recursive equation for the infinite-time dual ruin probability, namely $\psi ^*(\cdot )$, given by

4.1 $$\begin{aligned} \psi ^*(u+1)&= p_0 \psi ^*(u)+ \sum _{j=1}^\infty p_j \psi ^*(u+j) \end{aligned}$$

4.2 $$\begin{aligned}&= \sum _{j=0}^\infty p_j \psi ^*(u+j), \end{aligned}$$

with boundary conditions $\psi ^*(0)=1$ and $\lim _{u\rightarrow \infty } \psi ^*(u)=0$.

Equation (4.1) is in the form of an infinite-order difference (recursive) type equation. Thus, by adopting the general methodology for solving difference equations, we search for a solution of the form

$$\begin{aligned} \psi ^*(u)=cA^u, \end{aligned}$$

where *c* and *A* are constants to be determined. Using the given boundary conditions for $\psi ^*(\cdot )$, it follows that the constant $c=1$ and $0\leqslant A<1$. That is, the general solution to the recursive Eq. (4.1) is of the form

4.3 $$\begin{aligned} \psi ^*(u)=A^u, \end{aligned}$$

for some $0\leqslant A<1$. Substituting the general solution, given in Eq. (4.3), into Eq. (4.1), yields

$$\begin{aligned} A^{u+1}= \sum _{j=0}^\infty p_j A^{u+j}, \qquad u=0,1,2, \ldots , \end{aligned}$$

from which, dividing through by $A^u$ and defining the probability generating function (p.g.f.) of $Y_1$ by $\tilde{p}(z)=\sum _{i=0}^\infty p_iz^i$, we obtain

4.4 $$\begin{aligned} A= \tilde{p}(A), \qquad 0\leqslant A < 1. \end{aligned}$$

That is, $0\leqslant A<1$ is a solution (if it exists) to the discrete-time dual analogue of Lundberg’s fundamental equation, given by

4.5 $$\begin{aligned} \gamma (z)=0, \end{aligned}$$

where $$\gamma (z):=\tilde{p}(z)-z$$.

### Proposition 1

*In the interval* [0, 1) *there exists a unique solution to the equation* $\tilde{p}(z)-z=0$.

### Proof

It follows from the properties of a p.g.f. that

$$\begin{aligned} \gamma (0)&=p_0 \geqslant 0, \\ \gamma '(0)&=p_1 -1 \leqslant 0, \\ \gamma (1)&=0, \\ \gamma '(1)&=\mathbb {E}(Y_1) -1> 0, \\ \gamma ''(z)&>0, \quad \forall z\in [0,1). \end{aligned}$$

**Fig. 3:**
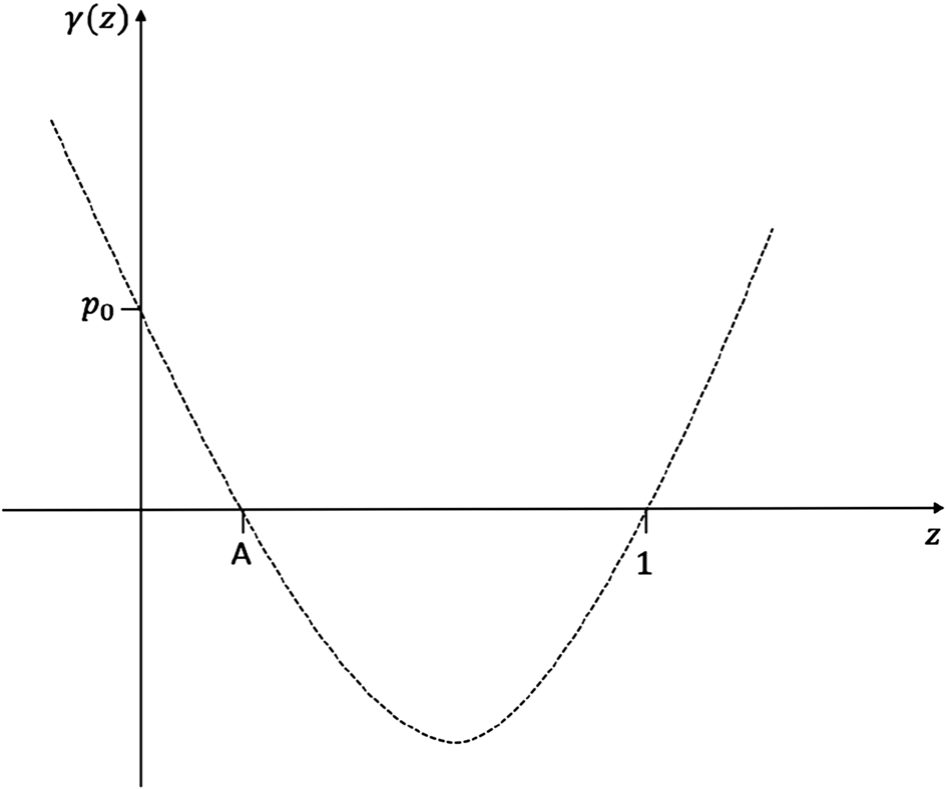
Graph of the function $\tilde{p}(z)-z$

From the above conditions, which show the characteristics of the function $\gamma (z):=\tilde{p}(z)-z$ (see Fig. 3), it follows that there exists a solution to $\gamma (z)=0$ at $z=1$ and a second solution $z=A$, which is unique in the interval [0, 1). $\square$

Hence, from Eqs. (4.3), (4.4) and Proposition 1, we obtain an expression for the infinite-time dual ruin probability, given by the following theorem.

### Theorem 3

*The infinite-time dual probability of ruin, namely* $\psi ^*(u)$ *for* $u \in \mathbb {N}$*, is given by*

4.6 $$\begin{aligned} \psi ^*(u) = A^u, \end{aligned}$$

*where A is the unique solution in the interval* [0, 1) *to the equation* $\tilde{p}(z)-z=0$*, with* $\tilde{p}(z)$ *the p.g.f. of* $Y_1$.

### Remark 2

We note that the p.g.f. $\tilde{p}(z)$ converges for all $|z| \leqslant 1$ and thus, in the interval $z\in [0,1]$ the p.g.f. exists (finite) for all probability distributions, i.e. light and heavy-tailed. Therefore, it follows that Theorem 3 holds for both light and heavy-tailed gain size distributions.

### Remark 3

Note that, for a general claim size distribution of $Y_i$, one cannot expect to observe the power law decay of heavy-tailed asymptotics, as is seen in the classic risk model, for the Parisian ruin probability $\psi _r^*(u)$. Indeed, recall that from Eq. (3.5) we have $\psi _r^*(u)= D \psi ^*(u)\le \psi ^*(u)$ for the constant $D=1-\frac{C^{-1}\sum _{k=1}^\infty a_k \phi ^*(k)}{1-C^{-1}\sum _{k=1}^\infty a_k \psi ^*(k)}$. Now, observing our discrete process, $R^*_n$, at the moments of claim arrivals, we can conclude that:

$$\begin{aligned} \psi ^*(u)=\mathbb {P}\left( \max _{n\ge 0}\sum _{i=1}^n (T_i-\tilde{Y}_i)\geqslant u\right) , \end{aligned}$$

where $\{T_i\}_{\{i=1,2\ldots \}}$ is a sequence of i.i.d. inter-arrival times independent of the renormalized sequence of i.i.d. claim sizes $\{\tilde{Y}_i\}_{\{i=1,2\ldots \}}$ with the law $\mathbb {P}(\tilde{Y_i}=k)=p_k/(1-p_0)$, for $k=1,2,\ldots$. In our model, the generic inter-arrival time, $T_i$, has the geometric distribution with the parameter $p_0$ and hence, is light-tailed. From the general theory of level crossing probabilities by random walks, see e.g. Theorem XIII.5.3 and Remark XIII.5.4 of [2] follows that the asymptotic tail of the ruin probability, $\psi ^*(u)$, always decays exponentially fast (this can also be seen from Theorem 3 and Remark 2). Therefore, the same concerns the Parisian ruin probability $\psi _r^*(u)$.

## Examples

In this section, in order to show the applicability of the above results, we consider the Binomial/Geometric model, as studied by Dickson [15], among others and Parisian ruin for the gambler’s ruin problem. In both cases, we derive an exact expression for the infinite-time dual probability of ruin, namely $\psi ^*(u)$ and consequently, from Theorem 3, we obtain an expression for the corresponding infinite-time Parisian ruin probability, $\psi ^*_r(u)$.

### Binomial/geometric model

In the Binomial/Geometric model, it is assumed that the gain size random variables $\{Y_i\}_{i \in \mathbb {N}^+}$ have the form $Y_i=I_i\cdot X_i$, where $I_i$ for $i\in \mathbb {N}^+$, are i.i.d. random variables following a Bernoulli distribution with parameter $b\in [0,1]$, i.e. $\mathbb {P}(I_1=1)=1-\mathbb {P}(I_1=0)=b$ and the random gain amount $\{X_k\}_{k\in \mathbb {N}^+}$ are i.i.d. random variables following a geometric distribution with parameter $(1-q)\in [0,1]$, i.e. $\mathbb {P}(Y_1=0)=p_0=1-b$ and $\mathbb {P}(Y_1=k)=p_k=bq^{k-1}(1-q)$ for $k\in \mathbb {N}^+$.

#### Lemma 3

*For* $u\in \mathbb {N}$ *, the infinite-time dual ruin probability,* $\psi ^*(u)$ *, in the binomial/geometric model, with parameters* $b\in [0,1]$ *and* $(1-q)\in [0,1]$ *such that* $b+q>1$ *, is given by*

5.1 $$\begin{aligned} \psi ^*(u)=\left( \frac{1-b}{q}\right) ^u. \end{aligned}$$

#### Proof

From Theorem 3, the infinite-time dual ruin probability, $\psi ^*(u)$, has the form $\psi ^*(u)=A^u$, where $0\leqslant A<1$, is the solution to $\gamma (z):=\tilde{p}(z)-z=0$, with

5.2 $$\begin{aligned} \tilde{p}(z)=1-b+b\tilde{q}(z), \end{aligned}$$

and $\tilde{q}(z)$ is the p.g.f. of a geometric random variable, which takes the form

5.3 $$\begin{aligned} \tilde{q}(z)=\frac{(1-q)z}{1-qz}. \end{aligned}$$

Combining Eqs. (5.2) and (5.3) and after some algebraic manipulations, Lundberg’s fundamental equation $\gamma (z)=0$, yields a quadratic equation of the form

$$\begin{aligned} z^2+k_1z+k_2=0, \end{aligned}$$

where

$$\begin{aligned} k_1&=\frac{b-1}{q}-1, \\ k_2&=\frac{1-b}{q}. \end{aligned}$$

The above quadratic equation has two roots $z_1=(1-b)/q$ and $z_2=1$. Finally, from the positive drift assumption in the the model set up, we have that $\mathbb {E}(Y_1)=b/(1-q)>1$, from which it follows that $b+q>1$ and the solution $z_1\in [0,1)$. Thus, we have $A=z_1$, since this solution is unique in the interval [0, 1) (see Proposition 1). $\square$

**Fig. 4:**
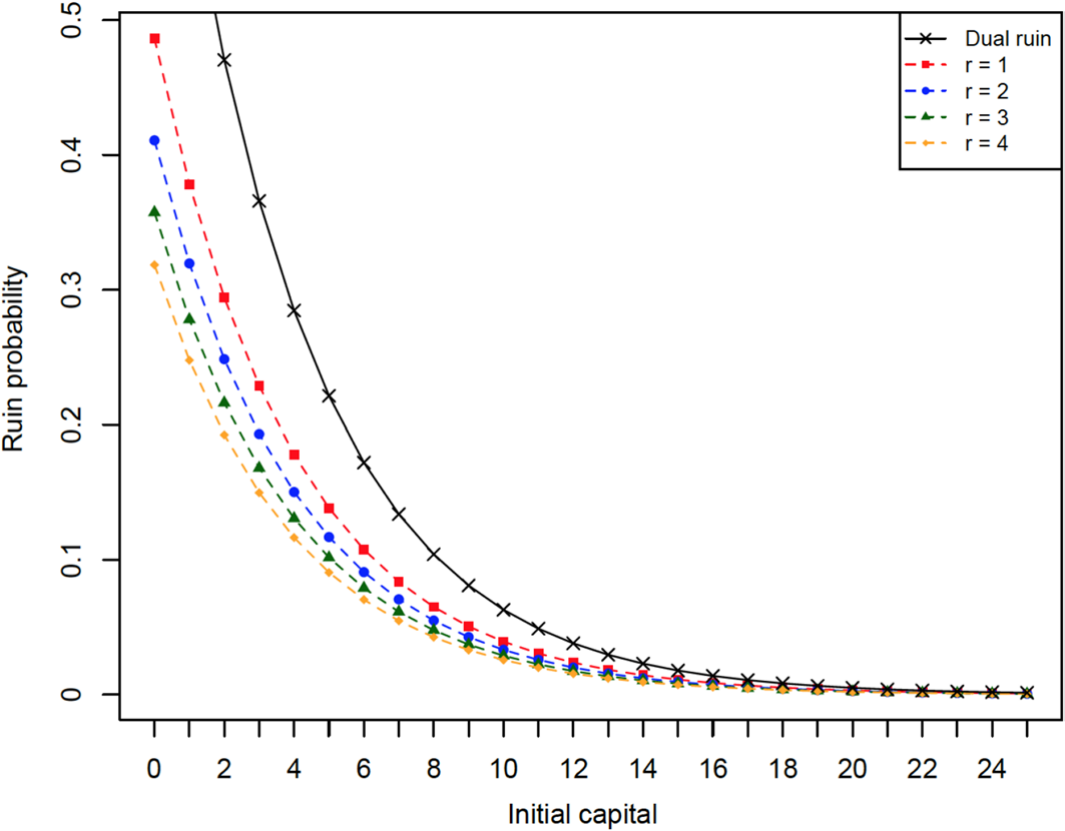
Plot of dual ruin and Parisian ruin probabilities for different values of *r*

To illustrate our results, in the binomial/geometric model, we consider the set of parameters, $b=0.3$, $q=0.9$. Then, the dual ruin probability and the Parisian ruin probabilities, for $r=1,2,3,4$, are given in the following plot (Fig. 4).

### Parisian ruin for the gambler’s ruin problem

In this subsection we consider a Parisian extension to the classic gambler’s ruin problem. In the gambler’s ruin model, a player makes a bet on the outcome of a random game, with a chance to double their bet with probability $b\in [0,1]$. The gambler continues to play the game, against an opponent with infinite funds, until he goes bankrupt, at which point he is declared as ruined (see [16]).

Mathematically, the gambler’s ruin model can be described by the discrete-time dual risk model, considered in the previous sections, with a loss probability $p_0=1-b$, corresponding win probability $p_2=b$ and $p_k=0$ otherwise. Further, in order to satisfy the net profit condition, and consequently avoid definite ruin over an infinite-time horizon, it follows that $b > 1/2$.

Under these assumptions Lundberg’s fundamental equation, $\gamma (z)=0$, produces a quadratic equation of the form

$$\begin{aligned} z^2 - \frac{1}{b}z+\frac{1-b}{b}=0, \end{aligned}$$

which has solutions $z_1=1$ and $z_2=\frac{1-b}{b}$. From the net profit condition, i.e. $b>1/2$, it follows that $z_2=\frac{1-b}{b} < 1$. Thus, from Theorem 3, we have that $A=\frac{1-b}{b}$ and the classic gambler’s ruin probability is given by

5.4 $$\begin{aligned} \psi ^*(u)=\left( \frac{1-b}{b}\right) ^u, \end{aligned}$$

as seen in [16]. Finally, from Theorem 2, the infinite-time Parisian ruin probability for the gambler’s ruin problem is given by the following proposition.

#### Proposition 2

*The infinite-time Parisian ruin probability to the gambler’s ruin problem, with win probability* $b > 1/2$*, is given by*

5.5 $$\begin{aligned} \psi _r^*(u)=\frac{1-bC_1}{1-(1-b)C_1}\left( \frac{1-b}{b}\right) ^{u+1} , \end{aligned}$$

where

5.6 $$\begin{aligned} C_1=\sum _{n=1}^{r}\, p_{n-1}^{*(n-1)} -\sum _{n=1}^{r} \sum _{i=2}^{n-1} \frac{1}{n-i} p_{n-1-i}^{*(n-i)}\,p_i^{*(i-1)}. \end{aligned}$$

#### Proof

Using the result of Theorem 2, and the form of the classic gambler’s ruin problem given by Eq. (5.4), it remains to find explicit expressions for the coefficients $a_k$, $k=1,\ldots , \infty$ and the constant $C^{-1}$.

Let us first consider the coefficients $a_k$, given by Eq. (3.6), of the form

$$\begin{aligned} a_k=\left( p_0 \mathbb {P}_{-1} (\tau ^- \leqslant r, R^*_{\tau ^-}=k) + p_{k+1} \right) . \end{aligned}$$

Recalling that in the gambler’s ruin problem the p.m.f’s of the positive gain sizes, i.e. $p_k=0$ for $k \ne 0,2$, it follows that only positive jumps of size $Y_i=2$, for $i\in \mathbb {N}^+$, can occur (with probability *b*) and thus, the joint distribution of recovery and the overshoot at the time of recovery, namely $\mathbb {P}_{-1} (\tau ^- \leqslant r, R^*_{\tau ^-}=k)=0$, for all $k\ne 0$. Thus, we have that, for $k=1,\ldots , \infty$, $a_k=p_{k+1}$ and it follows

$$\begin{aligned} a_k={\left\{ \begin{array}{ll} b, \qquad &{}k=1, \\ 0 \qquad &{}\mathrm{{otherwise}}. \end{array}\right. } \end{aligned}$$

Substituting this into the result of Theorem 2 and after some algebraic manipulations, we obtain

$$\begin{aligned} \psi _r^*(u)=\frac{C-b}{C-(1-b)}\left( \frac{1-b}{b} \right) ^u, \end{aligned}$$

where $$C=1-(1-b) \mathbb {P}_{-1}(\tau ^- \leqslant r, R^*_{\tau ^-}=0)$$.

Finally, by setting $z=0$ in Eq. (2.5) and noticing that, since $p_k=0$, for $k=3,4,\ldots$, only the term $j=n-1$ remains in both summation terms, we obtain

$$\begin{aligned} \mathbb {P}_{-1}(\tau ^- \leqslant r, R^*_{\tau ^-}=0)=b \left( \sum _{n=1}^{r}\, p_{n-1}^{*(n-1)} -\sum _{n=1}^{r} \sum _{i=2}^{n-1} \frac{1}{n-i} p_{n-1-i}^{*(n-i)}\,p_i^{*(i-1)}\right) , \end{aligned}$$

and it follows that $C=1-b(1-b)C_1$, where $C_1$ is given by Eq. (5.6). Finally, the result follows after some algebraic manipulations. $\square$

## References

[1] Albrecher H, Badescu A, Landriault D (2008). On the dual risk model with tax payments. Insur Math Econ.

[2] Asmussen S (2003). Applied probability and queues.

[3] Asmussen S, Albrecher H (2010). Ruin probabilities.

[4] Avanzi B, Gerber HU, Shiu ES (2007). Optimal dividends in the dual model. Insur Math Econ.

[5] Bergel AI, Rodríguez-Martínez EV, Egídio dos Reis AD (2016) On dividends in the phase-type dual risk model. Scand Actuar J. 10.1080/03461238.2016.1252944

[6] Boudreault M, Cossette H, Landriault D, Marceau E (2006). On a risk model with dependence between interclaim arrivals and claim sizes. Scand Actuar J.

[7] Cheng S, Gerber HU, Shiu ES (2000). Discounted probabilities and ruin theory in the compound binomial model. Insur Math Econ.

[8] Chesney M, Jeanblanc-Picque M, Yor M (1997). Brownian excursions and Parisian barrier options. Adv Appl Prob.

[9] Cheung EC, Drekic S (2008). Dividend moments in the dual risk model: exact and approximate approaches. Astin Bull.

[10] Cossette H, Landriault D, Marceau É (2003). Ruin probabilities in the compound Markov binomial model. Scand Actuar J.

[11] Cossette H, Landriault D, Marceau É (2004). Exact expressions and upper bound for ruin probabilities in the compound Markov binomial model. Insur Math Econ.

[12] Czarna I, Palmowski Z (2011). Ruin probability with Parisian delay for a spectrally negative Lévy risk process. J Appl Prob.

[13] Czarna I, Palmowski Z, Świa̧tek P (2016). Discrete time ruin probability with Parisian delay. Scand Actuar J.

[14] Dassios A, Wu S (2008) Parisian ruin with exponential claims. Department of Statistics, London School of Economics and Political Science, London, UK. **(Unpublished)**

[15] Dickson DC (1994). Some comments on the compound binomial model. Astin Bull.

[16] Feller W (1968). An introduction to probability theory and its applications.

[17] Gerber HU (1988). Mathematical fun with the compound binomial process. Astin Bull.

[18] Lefèvre C, Loisel S (2008). On finite-time ruin probabilities for classical risk models. Scand Actuar J.

[19] Li S, Sendova KP (2013). The finite-time ruin probability under the compound binomial risk model. Eur Actuar J.

[20] Li S, Garrido J (2002) On the time value of ruin in the discrete time risk model. Working paper 02-18, Business Economics, University Carlos III of Madrid

[21] Li S, Lu Y, Garrido J (2009). A review of discrete-time risk models. Rev Real Acad Cienc Exactas Fis Nat Ser A Mat.

[22] Loeffen R, Czarna I, Palmowski Z (2013). Parisian ruin probability for spectrally negative Lévy processes. Bernoulli.

[23] Michel R (1989). Representation of a time-discrete probability of eventual ruin. Insur Math Econ.

[24] Ng AC (2009). On a dual model with a dividend threshold. Insur Math Econ.

[25] Pavlova KP, Willmot GE (2004). The discrete stationary renewal risk model and the Gerber–Shiu discounted penalty function. Insur Math Econ.

[26] Shiu ES (1989). The probability of eventual ruin in the compound binomial model. Astin Bul.

[27] Takács L, Takács LM (1967). Combinatorial methods in the theory of stochastic processes.

[28] Willmot GE (1993). Ruin probabilities in the compound binomial model. Insur Math Econ.

[29] Wu X, Li S (2009). On the discounted penalty function in a discrete time renewal risk model with general inter-claim times. Scand Actuar J.

[30] Yuen K-C, Guo J (2006). Some results on the compound Markov binomial model. Scand Actuar J.

